# Anaerobic treatment of residuals from tanks transporting food and fodder

**DOI:** 10.1007/s11356-018-3876-z

**Published:** 2018-12-13

**Authors:** Van Than Nguyen, Erik Beyer, Jan Neumann, Dirk Awe, Wolfgang Pfeiffer, Jens Tränckner

**Affiliations:** 1 Faculty of Agricultural and Environmental Sciences, University of Rostock, Justus-von-Liebig-Weg 6, 18059 Rostock, Germany; 2 Department of Mechanical/Process and Environmental Engineering, University of Wismar, Philipp-Müller-Straße 14, 23966 Wismar, Germany; 3 TS-Clean Tank- und Siloreinigung Neumann GmbH, Ahorn Straße 9, 19288 Fahrbinde, Germany; 4 Rotaria Energie und Umwelttechnik GmbH, Kirchweg 21, 18230 Rerik, Germany

**Keywords:** Wastewater from the cleaning of tank cars transporting food and fodder, Anaerobic treatment, Biogas plant, Physiochemical model of anaerobic digestion

## Abstract

The anaerobic digestion of wastewater from the cleaning of tank cars transporting food and fodder was investigated in both bench and pilot scales with a single-stage, mesophilic (39 °C), completely mixed process. The promising results lead to the planning and building of a 1200-m^3^ full-scale biogas plant at TS-Clean cleaning station in Fahrbinde, Germany. Due to softened water used in the cleaning of the car tanks, the alkalinity in the digester decreased as predicted by the physicochemical model developed for this treatment process. The model showed that 2.4 kg NaHCO_3_/m^3^ of wastewater has to be added in order to control digester pH at 7.2 and to maintain the digester alkalinity at 3.1 g CaCO_3_/L. In a laboratory study, the decrease of alkalinity caused a volatile organic acids accumulation and pH drop below the optimal range. In this case, if chemical buffering was not added into the digester, the digester deteriorated. In a 3-year investigation, we confirmed that the strongly polluted WW from the cleaning of tank cars transporting food and fodder is suitable for an anaerobic treatment if the organic loading rate is controlled below 4 kg COD/m^3^/day, digester alkalinity is adjusted by NaHCO_3_, and micronutrients are added despite constant considerable variations in strength and composition of the wastewater. A biogas yield of 35–45 m^3^ CH_4_/m^3^ of wastewater and a COD elimination of 80–90% were achieved in bench- and pilot-scale experiments and are achieved in the full-scale biogas plant. The full-scale biogas plant is working stable with a biogas yield of 68 m^3^ biogas/m^3^ of wastewater.

## Introduction

Food and fodder like chocolate, cacao, sugar, milk, fruit juice, starch, different types of oils, and glycerol in the form of liquid, paste, or powder are transported in tank cars. The tanks require regular cleaning and disinfection. In Europe, about 300,000 tons of food and fodder are transported daily by tank cars. About 40,000–50,000 food and fodder transport containers are cleaned and disinfected daily in approximately 1600 cleaning stations (Philipowski 2016). Germany has some 100 cleaning stations.

The cleaning process consists in case of a strong pollution of the tanks in mechanical removal of remains and/or pre-cleaning with 160 °C steam. All tanks are cleaned with 85 °C hot soapy water and are finally rinsed with water. In all cleaning processes, softened water is used. Pre-cleaning and washing of considerably polluted tanks generate strongly polluted wastewater. Washing of only moderately polluted tanks and rinsing generates moderately polluted wastewater. The strongly and the moderately polluted wastewater (WW) is mostly not collected separately and are often discharged to the communal wastewater treatment plant (WWTP) without any pre-treatment. Sometimes, however, the wastewater is pre-treated in order to meet the standards for indirect discharge. Pre-treatment processes applied are mostly mechanical processes like flocculation/sedimentation and/or aerobic biological treatment processes (Philipowski 2008; Rudolph et al. 1995; Röhr and Müßig 2006).

**Fig. 1:**
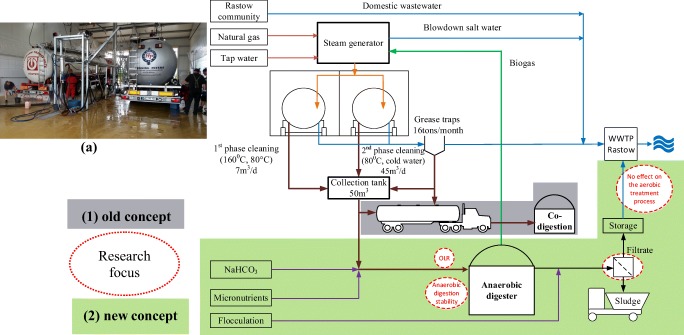
Cleaning lanes (a), scheme of old (1) and new (2) concept for the WW treatment at TS-Clean plant in Fahrbinde

TS-Clean company is operating three cleaning stations in Germany cleaning tank cars transporting food and fodder. At TS-Clean sites, strongly polluted WW is collected separately and discharged into an equalization tank. At Fahrbinde site, weekly, 35 m^3^ of strongly polluted WW for the cleaning of some 200 tank cars was transported to a biogas plant and later to a WWTP for co-digestion. The moderately polluted WW is discharged through a grease trap to the municipal WWTP. TS-Clean company had the idea to treat the strongly polluted WW from all three sites in Fahrbinde in order to produce biogas substituting natural gas used in the steam generator and thus reducing costs for natural gas and the wastewater disposal. The schemes of the old and the new concept for the WW treatment processes are shown in Fig. 1.

In the relevant technical literature, no information on the anaerobic digestion of WW from the cleaning of tank cars could be found. Due to that reason, the anaerobic digestion of this WW was investigated in bench- (3 L) and pilot-scale (500 L), before a full-scale digester was planned and built in Fahrbinde. In the investigation, the digester effluent was flocculated and filtrated and the filtrate treated in a sequencing batch reactor in order to confirm that a stable aerobic post-treatment meeting the direct discharge standards of the effluent of the anaerobic treatment is possible.

Preliminary experiments showed that the anaerobic digester operated well for a long time; however, the biogas production and chemical oxygen demand (COD) removal efficiency suddenly dropped after some 100 days of operation and the digester failed (data not shown). The sudden failure after such a long time of operation was considered to be the result of the softened water used in the cleaning, the deficiency of trace metals, maybe in combination with organic overloading, and insufficient alkalinity.

Relevant technical literature, reports that micro- and macronutrients play an important role for the growth, chemical reactions, and enzyme activity in the anaerobic digestion process (Choong et al. 2016; Mao et al. 2015). Recently, many researchers have reported deficits of micronutrients in anaerobic digesters treating food waste or energy crops with negative effects on biogas production and process stability. In digesters using sludge or manure as a substrate, trace elements however are present in abundant concentrations (Facchin et al. 2013; Schattauer et al. 2011). Food or similar waste is often found to be low in some metal ions; that can cause the anaerobic digester to fail. Among the trace elements (Co, Mo, Ni, Fe), Fe was identified as most effective for stabilizing anaerobic digesters (Zhang and Jahng 2012). The results also confirmed that supplemented trace metals enhance the biogas production and process stability of anaerobic digesters. With trace elements added, organic acids remained at low concentration, and pH maintained stable. For this reason, trace metal concentrations were monitored in our study in order to avoid safely trace metal deficits. In our study, micronutrients from ISF-Schaumann-Bioenergy were dosed as done in energy crop biogas plants.

Organic loading rate (OLR) is an important parameter in the anaerobic digestion process for COD removal efficiency and process stability. In technical literature, OLR in anaerobic digestion is reported in a wide range from 1 to 19 kg COD/m^3^/day (Jang et al. 2013; Jeganathan et al. 2006; Nagao et al. 2012). In a single-stage continuous digestion process, the OLR is typically in the range of 1.6–7.29 kg COD/m^3^/day. A too high OLR or a too dramatic increase in OLR can provoke an accumulation of volatile fatty acids (VFA) (Kleyböcker et al. 2012; Li et al. 2014). This imbalance of the digestion process can eventually cause a digester deterioration. In order to avoid imbalances and failures of the digestion process, the OLR has to be controlled to be below 3 kg COD/m^3^/day for anaerobic digestion of food waste (Xu et al. 2018). In our case, due to the highly polluted WW from the cleaning of tank cars transporting food and fodder varying constantly considerably in strength and composition, OLR control needs to be focused on.

In addition, due to softened water used in the cleaning of the tank cars, insufficient alkalinity, which might cause a digestion process imbalance, was studied with a physicochemical model in order investigate the necessity of an addition of NaHCO_3_. Relevant technical literature confirmed that addition of buffering chemicals like NaHCO_3_, Na_2_CO_3_, and NaOH or others like lime mud can enhance the digestion performance of food waste, synthetic wastewater, and kitchen waste (Chen et al. 2015; Gao et al. 2015; Silva et al. 2015; Zhang et al. 2014). In an agricultural biogas plant in Germany, the addition of sodium bicarbonate was used to control the pH and to stabilize the biological process resulting in an increased yield of methane (Burgstaler et al. 2011). In our study, the effect of an addition of NaHCO_3_ on the stability of the digestion process was also focused on.

In the WW-laboratory of the University of Wismar in cooperation with the University of Rostock, an anaerobic pre- and aerobic post-treatment process of the strongly polluted WW was investigated in bench and pilot scales. The purpose of the investigation was to find out if the anaerobic treatment process was stable in spite of WW continuously varying in strength and composition and if the effluent of the treatment would fulfill indirect discharge standards and be susceptible to aerobic post-treatment fulfilling direct discharge standards. In this paper, we are focusing on the anaerobic treatment of the highly polluted WW from the cleaning of tank cars transporting food and fodder. The anaerobic digestion process was conducted in a single-stage, mesophilic, and completely mixed process. In order to maintain the digestion process stable, the effect of OLR and the addition of NaHCO_3_ were investigated. On the basis of the promising results of the experiments, a full-scale anaerobic treatment plant was built at the tank car cleaning station of TS-Clean in Fahrbinde.

## Materials and methods

### Anaerobic digestion model

Due to the softened water used in the cleaning of the tank cars, it was anticipated that a deficit in buffer capacity might be a problem for the anaerobic treatment. Therefore, a physicochemical model for the steady-state anaerobic digestion of this WW was developed in order to show the relation of pH and total inorganic carbon (TIC). The model also demonstrates the interrelationship of the parameters pH, volatile organic acids (VOA), TIC, biogas production, and biogas composition. It helps the operator to control the process, to recognize beginning process imbalances, and to calculate the requirement of NaHCO_3_ addition for maintaining the pH and the process stable. The physicochemical model is based on the CO_2_ absorption equilibrium (Henry’s law) and the chemical equilibrium for ammonia, carbonic, and phosphoric acid and the balance of the ion charges. The alkalinity equation was adapted from Rittmann and McCarty (2012).

**Fig. 2:**
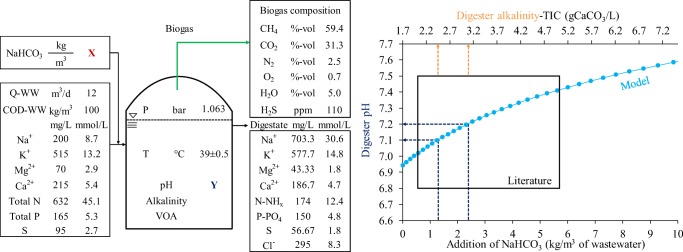
Steady-state physicochemical model for anaerobic treatment of strongly polluted WW

Figure 2 shows the physicochemical model and the results of the model calculations for the relationship of digester pH and alkalinity for normal CO_2_ concentrations in the biogas of 30–35%. The ion concentrations used in the model calculations are based on three independent measurements of the digestate performed for safeguarding sufficient concentrations of micronutrients. In order to maintain the digester pH = 7.2–7.3, the alkalinity has to be in the range of 3.0–4.0 g CaCO_3_/L. At low alkalinity, the digester pH can drop below 7.0. The alkalinity recommended for a stable digestion process is in the range of 1.5–5 g CaCO_3_/L. An alkalinity > 5 g CaCO_3_/L makes the digester pH insensitive to increasing VOA concentrations (Rittmann and McCarty 2012). An excessive alkalinity is therefore generating increasing operation costs without any benefit. The model results show that the addition of NaHCO_3_ is crucial for maintaining the digester pH in the optimal range. With no addition of NaHCO_3_, digester pH shall fall to even below 6.9. Based on the simulation, 2.4 kg NaHCO_3_/m^3^ WW has to be added in order to maintain digester pH at 7.2.

### Wastewater, seeding sludge, micronutrients, buffering chemicals

The highly polluted WW from the cleaning of tank cars transporting food and fodder is separately collected at TS-Clean plant Fahrbinde in a tank for a period of 1 week and is equalized and a 30- to 200-L sample was transported to our laboratory weekly. The WW chiefly contains fat (rape oil, palm oil, and cooking oil), protein (milk), carbohydrates (glucose, chocolate, fruit juice), and glycerol. The percentage of fats, carbohydrates, proteins, glycerol, and yeast is calculated based on the number of trucks, which are cleaned with the respective loads considering the degree of pollution according to the impression of the cleaner with factors of 1, 2, and 3 for slight, moderate, and strong pollution of the tanks. The analysis of some 100 different WWs in 2 years showed that the WW is complex, is highly polluted, and varies considerably in composition. The COD is in the range of 32–243 g/L. Due to the rapid acidification of the highly polluted WW, the pH is < 5. Corresponding to the number of trucks with loads rich in lipids (46%), the WW has an average COD/VS ratio of 2.2. The characteristic of the WW is shown in Table 1.

**Table 1:** The characteristic of the wastewater (mixtures of 1 week)

Parameter	Average	Ranges	Unit
pH	3.34	2.98–4.48	–
COD	96.50	31–243	g/L
Total solids (TS)	4.56	1.4–11.2	%
Volatile solids (VS)	4.33	1.3–10.8	%
Total Nitrogen	632	340–1000	mg/L
Total Phosphor	165	130–200	mg/L
Fats	46.1	21.1–58.1	%
Proteins	7.6	1.6–22.8	%
Carbohydrates	30.3	19–44	%
Yeast	5.8	0–16	%
Glycerol	6.1	0–16	%
Others*	3.9	0–11	%
Ca^2+^	0.215	0.20–0.23	g/L
Mg^2+^	0.07		g/L
K^+^	0.515	0.49–0.54	g/L
Na^+^	0.20	0.18–0.22	g/L

*mainly coffee and salt

The inoculum used for our research was taken from the pressure side of the recycle sludge pump of the 3.600 m^3^ mesophilic anaerobic sewage sludge digester of the WWTP Wismar, Germany. For the bench-scale experiments, an inoculum volume of 1.6 L sludge was used. The sludge stayed in a water bath at 39 ± 1 °C 3 to 4 days for degassing before the WW was added. In pilot-scale experiments, the digesters were filled completely with digested sewage sludge.

Micronutrients were added to the anaerobic digesters in order to compensate any deficit of trace metals of the wastewater. The nutrients and micronutrients were supplied by ISF-Schaumann-Bioenergy, Germany. The nutrient solution was added to the anaerobic digester corresponding to the COD load (0.19 g/kg COD of WW) as suggested by ISF-Schaumann-Bioenergy. The solution contained the trace elements copper (Cu), nickel (Ni), zinc (Zn), iron (Fe), boron (Bo), cobalt (Co), manganese (Mn), molybdenum (Mo), selenium (Se), and tungsten (W). Sodium hydrogen carbonate (NaHCO_3_) was added in order to stabilize the buffer capacity of the anaerobic digestion.

### Anaerobic digestion experiments set-up and procedures

The anaerobic digestion of the highly polluted WW from the cleaning of tank cars transporting food and fodder was investigated in bench- (3-L total volume) and pilot-scale (500-L total volume) single-stage, mesophilic, intermittent (bench scale), and completely (pilot scale) mixed reactors.

#### Bench-scale digesters

**Fig. 3:**
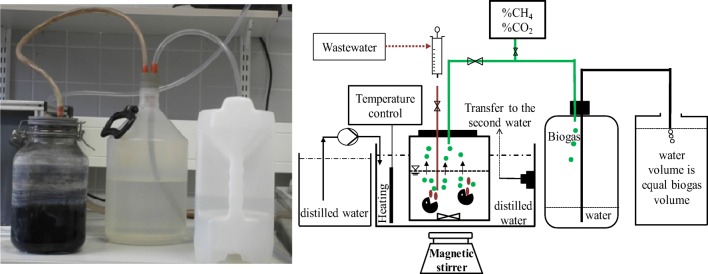
Schemes and photos of bench-scale digesters

Four glass digesters (B9, B10, B11, B12) with a working volume of 1.6 L were operated at 39 ± 1 °C. Photo and scheme of the experimental set-up of the bench-scale digesters are shown in Fig. 3. The feeding of the digesters was done manually 6 days a week. The feeding volume was 25–70 mL/day, resulting in hydraulic retention times (HRT) of 22–64 days. The digesters were shaken two to four times per day for mixing. The biogas production was measured daily. The water volume in the reception vessel is the volume of the biogas produced in the digester. Temperature and pressure correction have not been made because they balance with an error of less than + 5%. The biogas composition was analyzed twice a week with a gas analyzer (SR2-DO Sewerin, Germany).

#### Pilot-scale digesters

**Fig. 4:**
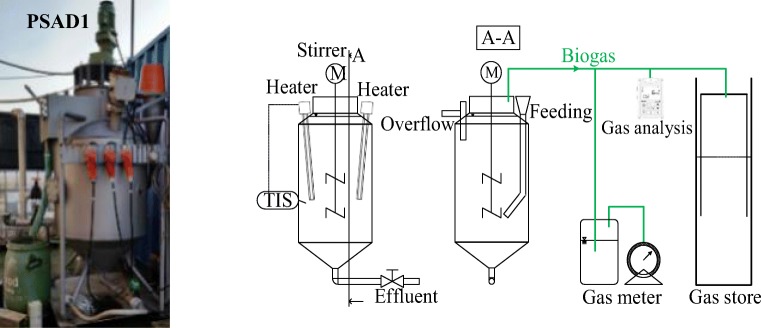
Schemes and photos of pilot-scale anaerobic digesters

Photos and schemes of the experimental set-up of the pilot-scale anaerobic digester 1(PSAD1) are shown in Fig. 4. The PSAD1 has a working volume of 450 L. The temperature was maintained at 39 ± 1 °C by electric heating. The digester was mixed with a three-arm propeller-stirrer (*Ø* 500 mm) 24 h/day with 100 rpm. The feeding volume was 5.8–31.6 L/day, with HRT of 14–77 days. Daily feeding was done manually and micronutrients well mixed with the WW before the WW was fed to the digester. Biogas volume was measured with a wet gas meter (Ritter, Germany) and biogas composition with infrared detectors (CH_4_ and CO_2_; SR2-DO Sewerin, Germany). Both were measured daily.

### Analytical methods

In the experiments pH, chemical oxygen demand (COD), total solids (TS), and volatile solids (VS) of the WW, biogas production and composition, pH, TS, VS, volatile organic acids (VOA), and total inorganic carbon (TIC) of the digestate were regularly measured.

VOA and TIC were measured with the FOS/TAC 2000 (Pronova, Germany). The FOS/TAC 2000 is using the Nordmann titration method. The effluent of the digester was filtrated through a paper filter before the analysis. VOA is measured in grams of acetic acid per liter. TIC is a measurement of the buffer capacity, and the unit is grams of CaCO_3_ per liter.

The pH of the effluent of the digester was measured immediately after sampling, in order to avoid pH changes due to a loss (desorption) of carbon dioxide. The pH was measured using a pH meter (Microprocessor pocket-pH 325, WTW, Germany). The COD was analyzed with NANOCOLOR tube tests (Macherey-Nagel, Germany). The COD tests mimic the DIN ISO 15705 procedure. The samples were heated to 148 °C for 60 minutes and chromium-VI to chromium-III reduction was measured by absorption of 620-nm light NANOCOLOR photometer 500D (Macherey-Nagel, Germany). TS and VS are analyzed according to the German Guideline DIN ISO 11465. Trace elements in the wastewater and digestate were analyzed with ICP (inductively coupled plasma) at the laboratory of the ISF-Schaumann Bioenergy, Germany.

## Results and discussions

### Bench-scale results

**Fig. 5:**
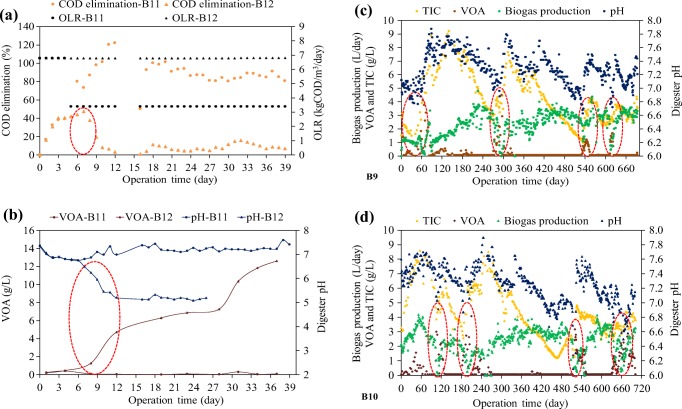
Results of bench-scale experiments (B9–B12) with provoked digester imbalances (**a**–**d**)

In digester B12, a process deterioration was provoked by overloading (Fig. 5a, b). COD degradation increased steadily to 80% in digester B11 and stayed stable at that level with initial OLR being reduced after 6 days from 6.8 to 3.5 kg COD/m^3^/day. Digester pH was stable at 7.15 and VOA concentration remained less than 300 mg/L. In digester B12 with a constant OLR of 6.8 kg COD/m^3^/day, COD degradation never surpassed 40%, VOA accumulated, and pH dropped. After 12 days of operation, COD degradation had decreased to less than 20%, VOA increased up to 13 g/L, and pH dropped to 5.5. Li et al. (2013) point out that in anaerobic digestion of food waste, VOA should not surpass 750 mg/L. When Li increased in his experiments, OLR from 6.7 to 8.4 kg VS/m^3^/day, the total VFA accumulated up to 8.4 g/L and pH dropped to 6.2. These results are comparable to our findings. Adaption to a higher OLR than 3.5 kg/m^3^/day with a moderate stepwise increase in OLR and adequate adaption times can however not be excluded.

Digesters B9 and B10 were operated with OLR in the range of 2 to 4 kg COD/m^3^/day. Without the addition of NaHCO_3_, digester alkalinity decreased constantly. pH followed this decrease but in a hardly measurable extent. Biogas production showed no indication unless critical conditions were surpassed. Then, biogas production showed a sharp decrease. VOA also did hardly react before critical conditions were surpassed. Then, the VOA concentration increased sharply and the anaerobic digestion was deteriorating. The decrease of alkalinity and pH and the effects of the decreasing biogas production and the VOA accumulation corresponded well with the simulation in the physicochemical model. Digester deteriorations are marked with red rings in Fig. 5c, d. The recovery of the process required the addition of NaHCO_3_ and a stop of feeding. This has to be avoided in full-scale operation. These results confirmed well the calculations of the physicochemical model. Lebuhn et al. (2014) reported that in a fermenter operated with maize silage initial alkalinity also decreased. Alkalinity decreased from 10 g CaCO_3_/L to 1 g CaCO_3_/L in the course of 1.5 years of operation. On the basis of their investigations, Burgstaler et al. (2011) came to the conclusion that NaHCO_3_ added to the fermenter stabilizes the pH and enhances the biogas yield in agricultural biogas plants in Germany.

### Pilot-scale results

**Fig. 6:**
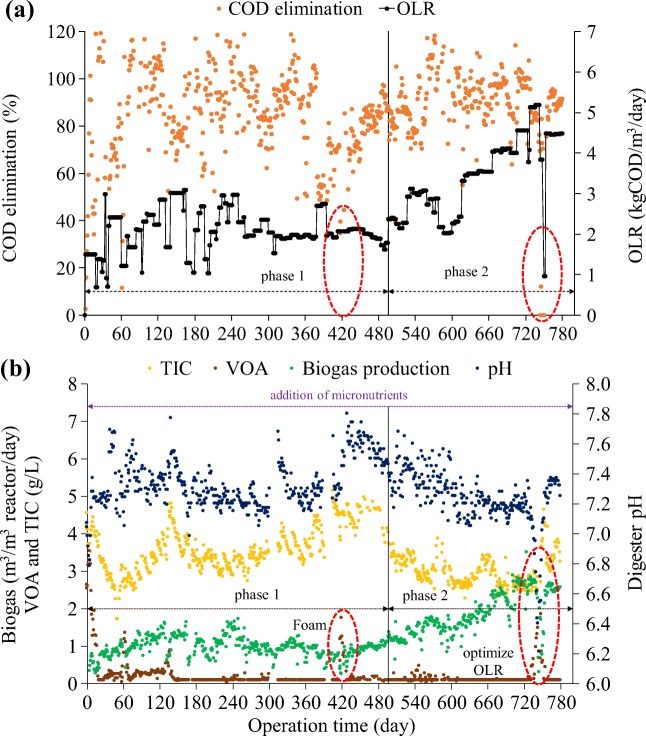
Results of pilot-scale experiments (PSAD1) with the stale digestion process (**a**, **b**)

Figure 6 shows the pilot-scale digester PSAD1 performance. Initially, in phase 1, alkalinity in PSAD1 decreased from 4.5 to 1.7 g CaCO_3_/L in the first 60 days. With alkalinity dropping below 2 g CaCO_3_/L, VOA concentration increased to about 1.35 g/L, pH slightly decreased to 7.05, and biogas production declined sharply. This is in good conformity with the bench-scale digester results. NaHCO_3_ was added to increase alkalinity and pH and the process performance recovered. 2.4 kg NaHCO_3_/m^3^ WW was then in phase 1 added in intervals for maintaining the alkalinity in the digester in the range 3–5 g CaCO_3_/L and the pH in the range of 7.1–7.3 in accordance with the physicochemical model developed for this process. OLR was controlled at 1.8–2.5 kg COD/m^3^/day. PSAD1 showed in this phase a stable COD elimination of approximately 88% and an average biogas production of 0.95 m^3^/m^3^ reactor/day and VOA was below 300 mg/L. VOA/TIC ratio was below 0.1 indicating a stable digestion process (Drosg 2013). An imbalance of the digestion process (day 420) occurred due to WW with an extreme high grease and oil content causing foam formation. The high grease concentration in the WW caused a considerable formation of creamy foam, an increase in VOA, and a decrease in biogas production. However, the foam layer could be destroyed by vigorously shaking the foam in a bench-scale digester. The dispersed foam was degraded in the bench-scale digester. The lesson learned from this incident is that WW with extreme fat and oil concentrations should be transferred to a hazard tank and be added diluted to the WW. An intense mixing of the surface volume could safeguard a re-immersion of creamy foam and creamy foam in the effluent should be recycled into the influent.

In phase 2, OLR was stepwise increased from 2 to 5.2 kg COD/m³/day in order to optimize OLR and feasibility. The biogas production increased from day 490 to day 720 from 0.95–2.7 m^3^ biogas/m^3^ digester volume/day rather parallel to the increase of the OLR from 2–4.5 kg COD/m^3^/day. The following increase from OLR 4.5 to 5.2 kg COD/m³/day on day 720 however lead to an accumulation of VOA from 0.1 to 1.9 g/L and a drop of pH from 7.2 to 6.4 on day 754. Then, the digester deteriorated, the feeding was stopped, and NaHCO_3_ was added in order to recover the digester again. The digesters recovered and operated well again with an OLR reduced to 4.5 kg COD/m^3^/day. The learned lesson is that in full-scale OLR shall not exceed 3 kg COD/m^3^/day in order to be on the safe side in case of troublesome WW with, i.e., high concentrations of fats and oils. During PSAD1 operation, the average NaHCO_3_ consumption was in the range of 2.17–3.15 kg NaHCO_3_/m^3^ of WW in accordance with the physiochemical model.

### Full-scale biogas plant performance

Based on the collected experience, we designed a full-scale anaerobic treatment plant for TS-Clean site Fahrbinde that was built by ROTARIA Energie und Umwelttechnik, Rerik, Germany. The full-scale plant is automated for remote control and operation.

**Fig. 7:**
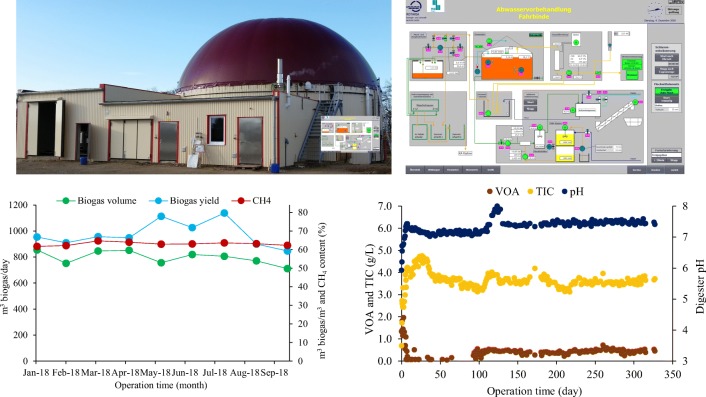
Photo, control screen, and performance data of full-scale biogas plant in Fahrbinde

Figure 7 shows a photo of the biogas plant and a snapshot of the process control and digester performance data. In November 2017, the 1200-m^3^ biogas plant was commissioned. The 300-m^3^ digested sludge from a cow manure biogas plant combined with the 700-m^3^ digested sludge from the anaerobic sewage sludge digester of the WWTP Wismar was used to start up the full-scale biogas plant. During the startup phase, the feeding volume of the wastewater was slowly increased to 10 m^3^/day in order to avoid an organic overloading.

The single-stage biogas plant is mixed intermittently and the temperature is controlled at 39 ± 0.5 °C. In order to control the digester pH and to maintain the digester alkalinity (TIC), sodium hydrogen bicarbonate is added to the digester according to the digestion model. Also, micronutrients are added according to ISF-Schaumann-Bioenergy suggestions, in order to avoid a deficit of trace elements. Daily pH, VOA, and TIC of the biogas effluent are monitored in order to control the biogas plant performance.

The monitored data show that digester pH is stable at 7.4. The digester pH is still slightly higher than expected due to digested cow manure and sewage sludge with high ammonia nitrogen concentrations used as inoculum. VOA is below 300 mg/L and alkalinity is controlled not to fall below 3.5 g CaCO_3_/L. The VOA/TIC ratio is well below 0.3, indicating a stable anaerobic digestion process (Drosg 2013). The full-scale data show a NaHCO_3_ consumption of 1.4 kg NaHCO_3_/m^3^ of wastewater. The NaHCO_3_ consumption is lower than calculated with the model. This is probably still due to the cow manure and domestic sewage sludge used as inoculum.

The daily biogas production of 12 m^3^ WW is approximately 800 m^3^, with 63% CH_4_ in the dry biogas. The biogas yield is 68 m^3^/m^3^ of WW. Substitution of natural gas through biogas saves some € 8.500 per month. Together with the savings in wastewater disposal, the return on investment is less than 5 years. The total solids in the biogas plant effluent are in the range 10–15 g/L. The effluent is flocculated and filtrated and the sludge is dewatered. The filtrate is discharged to the local WWTP Rastow. Eurofloc M-7 agent (Aquaplan, Germany) is used to floc the digester effluent (dosing 0.5% vol.). Flocculation, filtration, and sludge dewatering are realized in a screw filter press. Sludge separation and dewatering process are working stable. The consumption of the polymer is 36 kg/ton dry matter. The polymer dosage is 100 L solution (0.5%)/m^3^ digester effluent. The TS of the approximately 2.6-tons/week sludge cake is 25.4%. The filtrate COD is 1–2 g/L, and the filtrate pH is 7.5. The filtrate is discharged indirectly as it can be treated together with domestic wastewater with no adverse effects.

## Conclusions

Based on the experimental data and the model calculations, a 1200-m^3^ biogas plant was built in TS-Clean cleaning station Fahrbinde for treating the highly polluted WW from the cleaning of tank cars. We learned that an equalization tank with the capacity of the WW of 1 week makes changes in strength and composition of the WW sufficiently smooth for anaerobic digestion, if OLR is not too high (OLR < 4 kg COD/m^3^/day), HRT is long enough (HRT > 50 days), and micronutrients are added. Sufficient alkalinity in the digester is essential for stabilizing the digestion process of highly polluted WW. Due to softened water used in the cleaning of the tanks, alkalinity in the digester decreases daily. The required addition of NaHCO_3_ for controlling pH = 7.2–7.3 and maintaining alkalinity around 3–4 g CaCO_3_/L was calculated with a physicochemical model. The calculations of the model coincide well with the results of the digestion experiments and full-scale biogas plant operation. The biogas plant results can be used as reference data for an efficient anaerobic treatment of WW from food-processing industry. Based on these results, we are looking forward to build more biogas plants at car tank cleaning stations in Germany and in Europe. The biogas plant operators are trained for monitoring and controlling the digestion process based on the model calculation and personal experiences. For avoiding a VOA accumulation and an insufficient alkalinity, daily measurements of pH, VOA, and alkalinity are advised.

In order to recognize digestion process imbalances as early as possible and take appropriate action for avoiding a process deterioration, the physicochemical model needs to be expanded to study the influence of variations in strength and composition of the wastewater in combination with a digestion process inhibition due to overloading or inhibiting by toxic compounds in the wastewater causing an increase of VOA concentration.
